# The Pathophysiology and New Advancements in the Pharmacologic and Exercise-Based Management of Heart Failure With Reduced Ejection Fraction: A Narrative Review

**DOI:** 10.7759/cureus.45719

**Published:** 2023-09-21

**Authors:** Snaiha I Narayan, Giselle V Terre, Rutvi Amin, Keshvi V Shanghavi, Gayathri Chandrashekar, Farhana Ghouse, Binish A Ahmad, Gowri N S, Christena Satram, Hamna A Majid, Danielle K Bayoro

**Affiliations:** 1 Department of Medicine, Trinity College Dublin, Dublin, IRL; 2 Department of Medicine, Universidad Iberoamericana (UNIBE), Santo Domingo, DOM; 3 Department of Medicine, Surat Municipal Institute of Medical Education and Research, Surat, IND; 4 Department of Medicine, Lokmanya Tilak Municipal Medical College and Sion Hospital, Mumbai, IND; 5 Department of Medicine, D Y Patil University, Navi Mumbai, IND; 6 Department of Medicine, Saint James School of Medicine, St. Vincent, VCT; 7 Department of Medicine, King Edward Medical University, Lahore, PAK; 8 Department of Medicine, Taras Shevchenko National University of Kyiv, Kyiv, UKR; 9 Department of Medicine, Lincoln American University, Georgetown, GUY; 10 Department of Medicine, Dow University of Health Sciences, Dow International Medical College, Karachi, PAK; 11 Department of Medicine, Medical University of the Americas, Nevis, KNA

**Keywords:** exercise-based therapy, pharmacologic therapy, cardio-renal pathophysiology, reduced ejection fraction, heart failure

## Abstract

Heart failure with reduced ejection fraction (HFrEF) is a clinical syndrome whose management has significantly evolved based on the pathophysiology and disease process. It is widely prevalent, has a relatively high mortality rate, and is comparatively more common in men than women. In HFrEF, the series of maladaptive processes that occur lead to an inability of the muscle of the left ventricle to pump blood efficiently and effectively, causing cardiac dysfunction. The neurohormonal and hemodynamic adaptations play a significant role in the advancement of the disease and are critical to guiding the treatment and management of HFrEF. The first-line therapy, which includes loop diuretics, β-blockers, angiotensin-converting enzyme inhibitors/angiotensin II receptor blockers, hydralazine/isosorbide-dinitrate, and mineralocorticoid receptor antagonists (MRAs), has been proven to provide symptomatic relief and decrease mortality and complications. The newly recommended drugs for guideline-based therapy, angiotensin receptor/neprilysin inhibitor (ARNI), sodium-glucose cotransporter 2 inhibitors, soluble guanylate cyclase, and myosin activators and modulators have also been shown to improve cardiac function, reverse cardiac remodeling, and reduce mortality rates. Recent studies have demonstrated that exercise-based therapy has resulted in an improved quality of life, exercise capacity, cardiac function, and decreased hospital readmission rates, but it has not had a considerable reduction in mortality rates. Combining multiple therapies alongside holistic advances such as exercise therapy may provide synergistic benefits, ultimately leading to improved outcomes for patients with HFrEF. Although first-line treatment, novel pharmacologic management, and exercise-based therapy have been shown to improve prognosis, the existing literature suggests a need for further studies evaluating the long-term effects of MRA and ARNI.

## Introduction and background

Heart failure is a condition that develops when the heart fails to fill with or eject an adequate amount of blood to supply the body’s needs [1,2]. This often results from a functional or structural heart disorder impairing ventricular filling or ejection of blood to the systemic circulation [2-4]. Different diseases and conditions can cause heart failure. The etiology of heart failure can be classified into the following four broad categories: structural abnormalities; physiologic causes, which include biochemical and humoral mechanisms; extrinsic causes; and genetics [2,4]. The most common primary cause of heart failure is ischemic heart disease, also known as coronary artery disease. The second most common cause is non-ischemic heart disease, which is not related to coronary artery disease [5,6].

Heart failure can also be classified based on symptoms and calculated left ventricular ejection fraction (LVEF). There are three categories of left ventricular dysfunction defined by the calculated ejection fraction, namely, heart failure with reduced ejection fraction (HFrEF) (<40%), heart failure with preserved ejection fraction (HFpEF) (>50%), and heart failure with mid-range ejection fraction (HFmrEF) (40-50%) [1,2,4].

HFrEF stems from the left ventricle’s impaired contraction, resulting in an ejection fraction of less than 30% in patients with severe left ventricle dysfunction, and 30-40% in patients with moderate left ventricle dysfunction [2,7]. The underlying mechanisms involve significant loss of cardiomyocytes, acutely (e.g., following myocardial infarction or myocarditis) or chronically (e.g., genetic mutations or valvular disease with cell death due to overload) [8,9]. Neuroendocrine activation plays a crucial role, with stimulation of the carotid sinus and aortic arch baroreceptors leading to vasopressin release and activation of the renin-angiotensin-aldosterone system (RAAS), and consequently, vasoconstriction, increased preload, and afterload contribute to chronic chamber dilatation and heart failure [10-12]. A comprehensive understanding of these intricacies is essential for developing effective management for HFrEF.

Heart failure affects approximately 6.5 million adults in the United States, and nearly 50% of the cases have reduced ejection fraction [13]. HFrEF affects more men than women, and it exhibits significant morbidity and mortality [1,14]. In the most recent years, there have been significant scientific breakthroughs in the management of HFrEF. However, the morbidity and mortality continue to be high, with a five-year survival rate of 25% after hospitalization [1,15].

HFrEF is a multifaceted clinical syndrome that necessitates a treatment approach that incorporates patient education, pharmacologic management, and surgical interventions [4]. In this narrative review, we will discuss the neurohumoral and hemodynamic characteristics of the pathophysiology of HFrEF and provide an overview of the first-line therapies and new advancements in its pharmacologic and exercise-based management. Our goal is to highlight the new management based on the pathophysiological pathways that lead to HFrEF and identify gaps in clinical evidence to guide future studies.

## Review

Pathophysiology of heart failure with reduced ejection fraction

Neurohumoral Responses

The pathophysiology of HFrEF is characterized by a reactive model that is caused by primary myocardial injury leading to systolic dysfunction. The resulting reduced cardiac output initiates a series of maladaptive processes that include neurohumoral alterations, which trigger the sympathetic nervous system (SNS) and the renin-angiotensin-aldosterone system (RAAS) [2,10].

**Figure 1:**
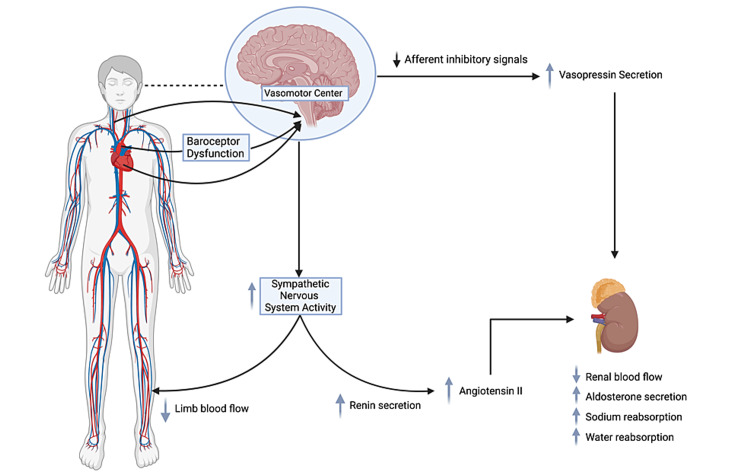
Schematic representation of the neurohormonal response to a reduction in cardiac output. Baroreceptor dysfunction triggers the vasomotor center causing an increase in sympathetic nervous system (SNS) activity and arginine-vasopressin secretion. The increased SNS activity causes peripheral vasoconstriction which aids in maintaining blood pressure. Along with the increased vasopressin, it leads to a decrease in renal blood flow, increased aldosterone secretion, and sodium and water reabsorption. The renal system also triggers an increase in renin secretion, which leads to an increase in angiotensin II (renin-angiotensin-aldosterone system pathway). Created by BioRender.com.

Following a fall in cardiac output, the SNS is one of the first systems to respond with a release of norepinephrine into circulation, affecting the kidneys, heart, and peripheral vasculature [11]. A decrease in cardiac output is sensed by baroreceptors in the left ventricle, arch of the aorta, and carotid sinus. In addition, the afferent nerves, from the arch of the aorta, send signals to the vasomotor center (VMC), resulting in the redistribution of blood flow to vital organs. In the peripheral vasculature, a heightened activity of the adrenergic nervous system stimulates the alpha-1 adrenergic receptors to induce peripheral arteriolar vasoconstriction, which aids in maintaining blood pressure. The neurohormonal activation increases the venous tone, which, in turn, accelerates the venous return to the heart, adding to the strain on the heart, or afterload. Furthermore, the SNS activates the beta-1 adrenergic receptors located on the juxtaglomerular apparatus of the kidneys to induce pro-renin release, which marks the initiation of the RAAS pathway [11,12] (Figure 1).

**Figure 2:**
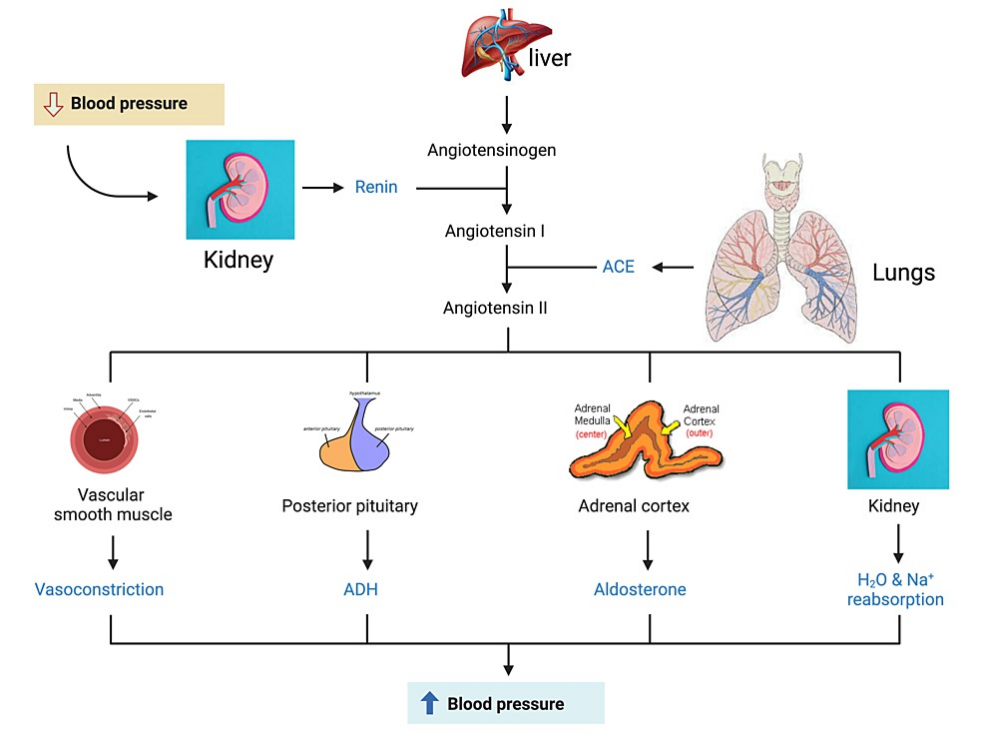
Schematic representation of the renin-angiotensin-aldosterone system activation in response to a reduction in blood pressure. The kidneys release renin, which converts angiotensinogen, released by the liver, to angiotensin I. The lungs release angiotensin-converting enzyme (ACE), which converts angiotensin I to angiotensin II. Angiotensin II, in turn, stimulates peripheral vasoconstriction, the posterior pituitary to release antidiuretic hormone (ADH), the adrenal cortex to release aldosterone, and the kidneys to increase water and sodium reabsorption. The result is a reactive increase in blood pressure. Created by BioRender.com.

The renin-angiotensin system (RAS) activity begins with pro-renin production, converted to renin via proteolytic activation. It can have either a direct or an indirect action on the kidneys. Renin is an aspartic protease that cleaves angiotensinogen systemically or locally to lead to the production of angiotensin 1. Angiotensin 1 is a precursor converted to angiotensin 2 via the angiotensin-converting enzyme (ACE) [11,16]. Angiotensin 2 acts on two major receptors, AT1 and AT2. Its action on AT1 in the zona glomerulosa of the adrenal glands results in the secretion of aldosterone, which mediates sodium and water retention. Angiotensin II also stimulates the brain’s thirst center and provokes the release of vasopressin from the posterior pituitary, increasing water retention in the body (Figure 2). Due to increasing osmolality, vasopressin is secreted, resulting in increased water retention to bring the osmolality back to normal. However, with respect to HFrEF, the vasopressin concentration is elevated. Due to this vasopressin release, hyponatremia results, along with increased endothelin production [16].

Activation of the SNS also results in a fall in the levels of atrial natriuretic peptide (ANP) and brain natriuretic peptide (BNP). ANP and BNP are among the most important RAAS counterregulatory hormones secreted in response to atrial stretch. They increase cGMP production, which causes increased renal excretion of water and sodium. In the setting of HFrEF, vasoconstriction develops which results in an increased intravascular volume and increased afterload [17].

Nitric Oxide-Soluble Guanylate Cyclase-cGMP Pathway

The nitric oxide (NO)-soluble guanylate cyclase (sGC)-cGMP signaling pathway is critical in blood vessel physiology [18]. Endogenous NO binds to the heme group of sGC in the smooth muscle leading to activation of the smooth muscle. This enzyme converts GTP to cGMP, leading to vasodilation, inhibition of platelet aggregation, and smooth muscle proliferation [17]. In the heart, natriuretic peptides activate this pathway, providing cardioprotective effects such as improved diastolic relaxation, coronary blood flow, and reduced hypertrophy, inflammation, and fibrosis. In HFrEF, reduced blood flow causes oxidative stress and inflammation, decreasing NO production and increasing degradation, as well as disrupting the NO-sGC-cGMP pathway. This dysregulation fails to counteract neurohormonal activation, worsening its effects [8,18].

Hemodynamic Adaptations and Cardiac Remodeling

**Figure 3:**
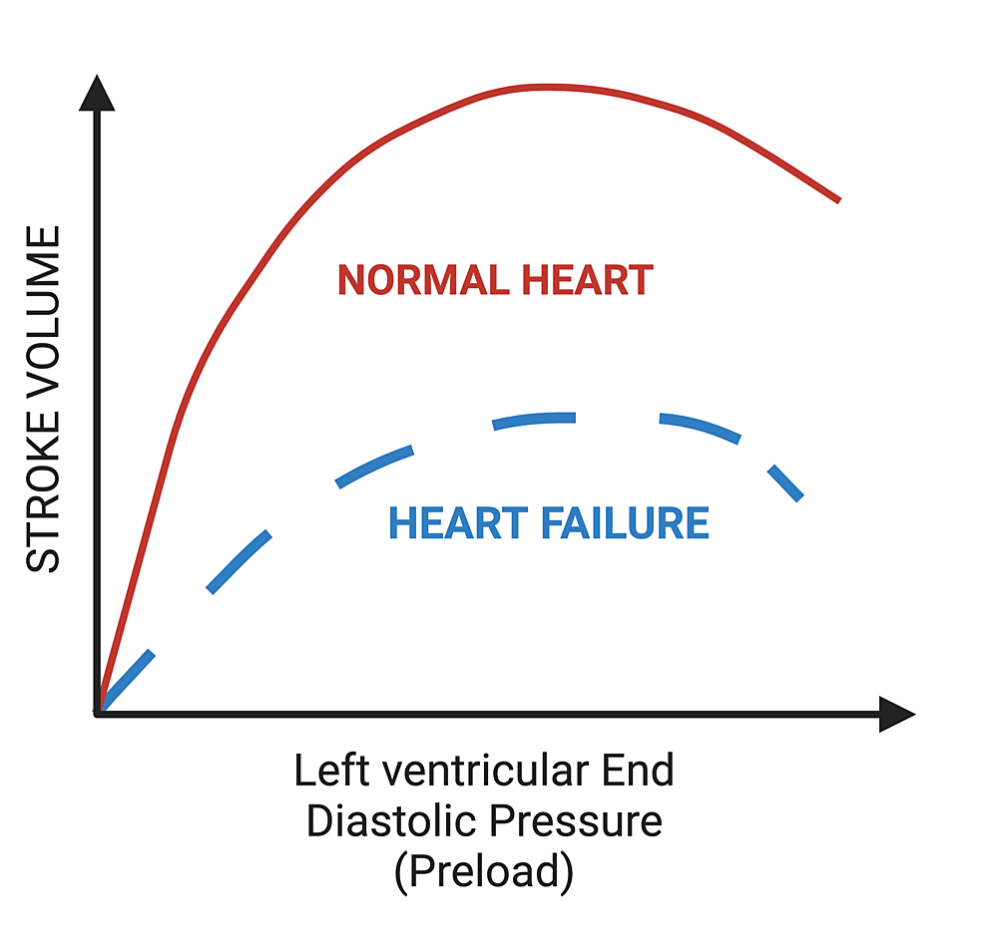
Frank-Starling mechanism that displays the response of the heart to a reduction in venous return. The Frank-Starling curve in heart failure exhibits a downward shift depicting that to increase contractility and stroke volume, there must be increased venous return and filling pressure. This explains the increase in fluid retention resulting from cardiac dysfunction. Created by BioRender.com.

**Figure 4:**
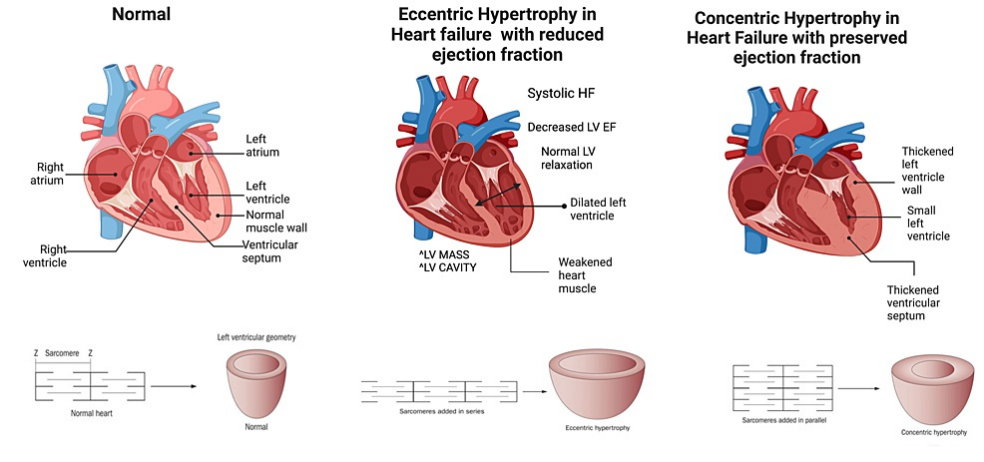
Cardiac remodeling in heart failure: eccentric hypertrophy and concentric hypertrophy in patients with heart failure compared to a normal heart. Eccentric and concentric hypertrophy are characterized by the arrangement of the sarcomeres. In heart failure with reduced ejection fraction, sarcomeres are added in a series rather than parallel as in concentric hypertrophy. Created by BioRender.com.

The hemodynamic adaptations in HFrEF are characterized by left ventricular dilatation, eccentric left ventricular hypertrophy, and abnormal systolic and diastolic function. Typically, during exercise, there is an increase in the body’s oxygen consumption, where the left ventricle raises its cardiac output to meet the body’s demands [2]. Increased cardiac output is typically achieved through elevation in heart rate and/or increased left ventricular stroke volume (SV). For patients with HFrEF to yield an adequate SV and cardiac output, they require a sizeable end-diastolic volume [9]. However, reduced left ventricular contractility in heart failure leads to reduced elastic recoil and abnormal filling, causing a downward shift in the Frank-Starling curve [19] (Figure 3). This results in a decreased SV and compensatory increase in end-diastolic pressure due to incomplete ventricular emptying. When the left ventricle of the heart cannot fill adequately and/or eject blood, hemodynamic overload results, and the heart becomes incapable of meeting the demands of the body’s tissues [8,9]. Over time, an increase in left ventricular volume leads to ventricular dilation, where new sarcomeres are added in series to existing sarcomeres, thus leading to eccentric hypertrophy and cardiac remodeling and dysfunction [20] (Figure 4).

First-line pharmacologic approach

The current management of HFrEF aims to provide symptomatic relief and decrease mortality and complications associated with the reduced ejection fraction. The conventional first-line therapy includes loop diuretics, RAS inhibitors (angiotensin-converting enzyme inhibitors (ACEis) and angiotensin II receptor blockers (ARBs)) or hydralazine/isosorbide-dinitrate, and a β-blocker [3,13]. These classes of drugs are associated with significant reductions in mortality and morbidity [3]. Loop diuretics reduce intravascular volume, thereby decreasing blood pressure. Hydralazine/isosorbide-dinitrate is a smooth muscle relaxant used as an alternative to ACEi/ARBs as tolerated.

Angiotensin-Converting Enzyme Inhibitors and Angiotensin II Receptor Blockers

ACEi/ARBs work by inhibiting the RAS system that, in sequence, lowers the blood pressure and afterload, addressing a significant pathophysiologic pattern of HFrEF. CHAMP-HF, a cohort study aimed at filling the gaps in the use and dosage of medication prescribed for outpatient treatment of HFrEF, observed the highest rate of hospitalization among patients receiving the lowest doses of ACEi/ARB [21]. Studies have shown that higher doses of ACEi/ARB therapy are directly proportional to reduced hospital stay and better prognosis. ACEi/ARBs have been proven to play a crucial role in the mainstay treatment of HFrEF while carefully monitoring blood pressure and serum potassium and creatinine levels [22].

β-Blockers

β-blockers work by blocking the sympathetic effect of beta-adrenergic receptors on the heart. According to numerous studies, β-blockers are considered the first-line therapy in compensated HFrEF. They work by inhibiting the SNS and the RAS with beneficial effects, including a decrease in heart rate and blood pressure and a reduction in the probability of subsequent arrhythmias and myocardial infarction. Extensive clinical trials have established that β-blockers limited heart failure hospitalization and improved survival [23,24]. According to COMET, a randomized control trial (RCT), selective β-blockers such as metoprolol and carvedilol were associated with a 40% and 34% reduction in mortality rate, respectively [23]. However, careful heart rate, blood pressure, and myocardial function evaluation are required during titration and after the target dose is reached [22].

Mineralocorticoid Receptor Antagonists

Mineralocorticoid receptor antagonists (MRAs) are considered the most unrecognized therapy in HFrEF despite significant evidence from various randomized trials in improving mortality [25,26]. A study based on the RALES and EMPHASIS-HF trials concluded that MRA therapy did not cause any significant decrease in blood pressure even when the baseline systolic blood pressure was low [26]. Apart from maintaining saltwater homeostasis, MR blockade helps reduce inflammation, which consequently reduces cardiovascular and renal damage. A randomized clinical trial based on data from the Cochrane Central Register of Controlled Trials, MEDLINE, and EMBASE concluded that the benefit of using MRA pharmacotherapy was significantly higher for HFrEF [27]. This regimen is introduced in patients with symptomatic chronic HFrEF already receiving β-blockers and ACEi/ARBs [25-27].

Ivabradine

Heart rate is a significant predictor of prognosis in heart failure. Ivabradine is added to the initial management of HFrEF once β-blockers are fully titrated and reach the target dose [28]. According to the 2016 American College of Cardiology/American Heart Association/Heart Failure Society of America (ACC/AHA/HFSA) guideline, ivabradine therapy in patients with HFrEF decreased the risk of heart failure hospitalization [29]. According to the SHIFT trial, patients who had a baseline heart rate of greater than 77 beats/minute, those who could not tolerate the up-titration, or for whom these agents were contraindicated had a significant reduction in hospitalization. Ivabradine acts on the current in the sinoatrial nodal activity and reduces the heart rate without affecting blood pressure. The SHIFT trial also concluded that 5% of the patients on ivabradine therapy had symptomatic bradycardia while 6% had asymptomatic bradycardia compared to 1% in the placebo group [28,30].

Novel pharmacologic therapies

While several treatment options are available for patients with HFrEF, adding new agents remains essential to improve the prognosis and manage the condition effectively. According to the 2022 AHA/ACC/HFSA guidelines, there are four recommended drugs for guideline-mediated therapy in HFrEF treatment. These include angiotensin receptor/neprilysin inhibitors (ARNIs), sodium-glucose cotransporter 2 (SGLT2) inhibitors, sGC modulators, and myosin activators [3]. Each of these drugs plays a distinct role in targeting the pathophysiology of HFrEF, and their inclusion in the treatment regimen is advised based on their proven efficacy and potential benefits for patients with HFrEF.

Angiotensin Receptor/Neprilysin Inhibitor

Sacubitril/valsartan, an inhibitor of angiotensin receptor-neprilysin, has demonstrated a reduction in the risk of cardiovascular death and hospitalization due to heart failure, as well as improved symptoms in patients with HFrEF [31]. Despite multiple drug regimens, mortality rates remained high among HFrEF patients. Consequently, in 2015, the US Food and Drug Administration approved a novel drug called sacubitril/valsartan, marketed as Entresto®, which combines an ARB with the neprilysin inhibitor prodrug sacubitril in a 1:1 ratio. By inhibiting neprilysin, sacubitril prevents the degradation of natriuretic peptides. Thus, this combination addresses two fundamental pathophysiological mechanisms, namely, activation of the RAAS and decreased sensitivity to natriuretic peptides [32]. Its recommended use is in place of ACEi/ARBs as tolerated and with other medications included in the first-line therapy of HFrEF [33].

One well-known clinical trial, PARADIGM-HF, compared sacubitril/valsartan to enalapril, an ACEi, and demonstrated a 20% reduction in relative risks for the combined primary endpoints of death and heart failure hospitalization. The study also examined mortality rates due to two leading causes: sudden death and ventricular arrhythmia. The study revealed a 22% reduction in the risk of resuscitated or non-resuscitated sudden deaths in patients treated with ARNI compared to enalapril [34,35]. ARNI also reduced sudden deaths by 50% in patients with an implantable cardioverter-defibrillator (ICD) [36]. These effects were not observed in patients treated solely with ACEis or ARBs. The effects of ARNI were analyzed in HFrEF and ICD for various conditions such as appropriate shocks, non-sustained ventricular tachycardia, supraventricular tachycardia, ventricular extra systolic load, and the percentage of biventricular pacing. The post-intervention analysis showed significant clinical improvement, decreased arrhythmic load, a five-point increase in ejection fraction, and reduced NT-proBNP levels. A direct correlation was observed between NT-proBNP levels and ventricular arrhythmic load [34,37].

In the PARADIGM-HF trial, the sacubitril/valsartan group showed 23% lesser rates of hospitalizations for worsening heart failure. The reduction was irrespective of the patient characteristics and decreased first and recurrent hospitalization [31]. The study revealed that patients using ARNIs experienced a reduced frequency of severe hyperkalemia compared to the alternative drug [34]. Moreover, when comparing the effects of both drugs on renal function, it was found that the benefits were twice as high in patients utilizing ARNIs [38]. Notably, the number of patients who had to discontinue ARNIs due to renal insufficiency was only half that of those taking enalapril [39]. An analysis was conducted to assess the drug’s impact on quality of life and functional capacity, yielding noteworthy improvements in both aspects [40]. Initiating ARNIs early has demonstrated significant advantages in both acute and chronic scenarios. These findings make the role of ARNI significant in treating patients with HFrEF as it reduces hospitalization and mortality from heart failure.

Sodium-Glucose Cotransporter 2 Inhibitors

SGLT2 inhibitors provide numerous advantages for patients with HFrEF. SGLT2 are sodium-glucose cotransporters expressed in the proximal convoluted tubules and are responsible for the reabsorption of 90% of the filtered glucose [41]. Thus, inhibiting SGLT2 promotes early natriuresis, leading to a reduction in plasma volume and subsequent lowering of blood pressure and tissue sodium levels. Numerous clinical trials, including EMPA-REG OUTCOME, CANVAS, DECLARE-TIMI 58, and VERTIS, have been conducted to evaluate the effectiveness of SGLT2 inhibitors in treating HF. These trials have demonstrated the ability of these drugs to not only decrease HF-related hospitalizations but also lower cardiovascular mortality rates. Notably, studies such as DAPA-HF and EMPEROR-Reduced have emphasized the significance of SGLT2 inhibitors as a fourth pillar in modifying the prognosis of HFrEF, thus complementing the gold standard treatment regimen [42].

SGLT2 inhibitors have exhibited effectiveness in patients with type 2 diabetes (T2D), who often experience cardiovascular complications, including heart failure. Clinical trials involving patients with and without T2D have shown that SGLT2 inhibitors improve cardiovascular outcomes and reduce associated risk factors in these individuals [43]. A recent study has identified newly diagnosed HFrEF patients hospitalized with heart failure as ideal candidates for SGLT2 inhibitors. Administration of these drugs to such patients has demonstrated a substantial reduction in clinical events within days and weeks. Additionally, SGLT2 inhibitors possess renal protective properties and diminish the risk of hyperkalemia, making them favorable for concurrent use with RAS inhibitors [44]. This can reduce mortality and morbidity in patients with or without T2D.

Soluble Guanylate Cyclase Stimulators

sGC stimulators, such as vericiguat, activate sGC independently of NO by binding to the enzyme’s non-heme side [45]. New drugs targeting the NO-sGC-cGMP signaling pathway have been crucial in addressing the impact of neurohormonal activation.

Two necessary clinical trials were conducted for vericiguat. The SOCRATES-REDUCED study demonstrated that vericiguat was well-tolerated, and higher doses were associated with a more significant decrease in NT-proBNP levels [46]. In the VICTORIA trial, vericiguat was linked to a reduced risk of hospitalization and death from cardiovascular events, showing promise for patients with HFrEF [47].

Modulators and Myosin Activators

Omecamtiv mecarbil is a cardiac myosin activator that aims to enhance sarcomere function and improve myocardial function [48-50]. In the COSMIC-HF study, omecamtiv mecarbil was administered to patients with HFrEF for 20 weeks. Substantial enhancements in systolic function, ejection time, ejection fraction, fractional shortening, and SV were noted [51-54]. The treatment also decreased NT-proBNP levels, heart rate, and neurohormonal activation. Notably, left ventricular diastolic and systolic dimensions and volumes showed significant improvement, indicating the drug’s potential to reverse cardiac remodeling. These findings suggest that omecamtiv mecarbil can potentially improve mortality rates and reduce hospitalizations in patients with HFrEF [55,56]. In contrast, the METEORIC-HF clinical trial, focused on chronic HFrEF patients, did not demonstrate any improvement in exercise capacity with the administration of omecamtiv mecarbil [57]. However, a separate trial GALACTIC-HF evaluating the drug’s efficacy and safety in patients with low kidney function, low blood pressure, and those receiving optimum medical, or device management revealed positive effects, with a hazard ratio of 0.80 (95% CI = 0.71-0.90) for the primary endpoint and 0.88 (95% CI = 0.75-1.03) for cardiovascular deaths in severe heart failure patients [58].

Effects of exercise-based therapy on mortality, hospitalization readmission rates, health-related quality of life, and cardiovascular function

The pathophysiology of the effects of exercise-based therapy in patients with HFrEF involves various processes and adaptations. Studies have shown that patients with HFrEF have a decreased cardiac and pulmonary reserve, skeletal muscle dysfunction, and autonomic dysfunction. These central and peripheral mechanisms contribute to the exercise intolerance observed in patients with heart failure, thus contributing to a poor quality of life, mortality, and hospitalizations [59].

Exercise training and regular physical activity have been shown to improve exercise tolerance and functional capacity in patients with heart failure by several mechanisms. Primarily, regular exercise training improves the autonomic dysfunction seen in HFrEF. It decreases sympathetic activity and thus reduces plasma catecholamine and angiotensin II levels. This consequently improves baroreflex and decreases the potentially harmful effects of both plasma catecholamines and angiotensin II. Concurrently, it increases parasympathetic activity thus improving heart rate variability (HRV) [59,60]. HRV refers to the degree of variability between consecutive heartbeats, or RR intervals. A low HRV is suggestive of autonomic dysfunction and is a predictor of mortality in patients with heart failure [59].

Exercise training also improves endothelial function by increasing the synthesis of NO synthase, which causes vasodilation of the endothelium. Decreased sympathetic activity as well as improved endothelial function cause a decrease in peripheral resistance. These resulting adaptations decrease cardiac afterload and, therefore, reduce myocardial oxygen demand, increase LVEF, and contribute to increased blood flow to the skeletal muscles [60,61].

Exercise has been shown to reduce inflammation by increasing anti-inflammatory cytokines such as interleukin (IL)-10 and reducing pro-inflammatory cytokines (e.g., tumor necrosis factor-alpha, IL-6, and IL-1β), markers of endothelium dysfunction and inflammatory mediators (e.g., vascular cell adhesion molecule, intercellular adhesion molecule, and monocyte chemoattractant protein-1) [60,62]. These changes have several positive effects on improving pulmonary ventilation, renal function, peripheral and coronary arterial remodeling, and contractile function as well as energy transfer of myocardial and skeletal muscle [60,63]. Furthermore, exercise therapy improves the structure and function of skeletal muscles, thereby improving skeletal muscle remodeling. The involved mechanism produces increased capillary density in the muscles, improved skeletal muscle fiber type distribution, and increased mitochondria concentration, which, in turn, increases oxidative phosphorylation. These combined changes result in improved LVEF and peak oxygen consumption (VO_2_) [64].

Notably, exercise and regular physical activity improve end-diastolic and end-systolic volumes and filling pressures, which lead to favorable changes in the structure of atria and ventricles, rendering them capable of supporting higher maximal heart rates, high systolic blood pressures, and increased ejection fractions during exercise, thus improving exercise intolerance [60].

Several studies have shown that exercise therapy improves quality of life and decreases hospitalization readmission rates. The results on the effect of mortality rates have been variable. For example, a review included RCTs that compared exercise therapy with a follow-up of six months or longer in adults over 18 years of age having either HFrEF or HFpEF concluded that exercise therapy reduces the risk of hospital readmissions regardless of the cause and of heart failure-specific readmissions in the short term (less than 12 months) and improves health-related quality of life (HrQOL). The study found no evidence of decreased mortality in the short term [65]. Another review, which included both HFpEF and HFrEF patients, concluded that there was a trend toward a decrease in all-cause mortality in RCTs with follow-ups of more than 12 months [66]. A systematic review and meta-analysis conducted by Bjarnason-Wehrens et al. found no association between exercise therapy and mortality and hospitalization readmission rates. However, the study did find an improvement in HrQOL [67].

A study conducted by Bjarson-Wehrens et al. concluded that a combination of aerobic and resistance training is better than aerobic training alone in a selected group of patients (patients with moderate-to-good left ventricular function, good cardiac performance capacity (more than five to six metabolic equivalents of oxygen consumption = 1.4 watt/kg body weight), no symptoms of angina pectoris or ST segment depression under continued maintenance of the medical therapy). According to Bjarson-Wehrens et al., resistance training positively impacts cardiovascular risk factors, cardiovascular function metabolism, and quality of life [67].

Between moderate-intensity continuous training (MICT) and high-intensity interval training (HIIT), daily MICT is recommended. A systematic review and meta-analysis conducted by Tucker et al. to study the effect of MICT, HIIT, and resistance training on left ventricular remodeling found that MICT decreases left ventricular remodeling with the most benefits seen with more than six months of MICT training. HIIT for two to three months was better than controls at improving LVEF but was not superior to MICT. Resistance training alone or in combination with aerobic training was not found to improve LVEF [68,69].

Discussion

HFrEF stems from the left ventricle’s impaired contraction, resulting in an ejection fraction of less than 40% [3]. The underlying mechanisms involve significant loss of cardiomyocytes, acutely (e.g., following myocardial infarction or myocarditis) or chronically (e.g., genetic mutations or valvular disease with cell death due to overload) [14,15]. Neuroendocrine activation plays a crucial role, with stimulation of the carotid sinus and aortic arch baroreceptors leading to vasopressin release and activation of the RAAS, and consequently, vasoconstriction, increased preload, and afterload contribute to chronic chamber dilatation and heart failure [8-10]. A comprehensive understanding of these intricacies is essential for developing effective treatments for HFrEF.

ACEis are the cornerstone of HFrEF treatment. These medications inhibit the RAAS, reducing vasoconstriction and afterload and ultimately improving cardiac function. Clinical trials, such as the large European study BIOSTAT-CHF, have shown the importance of appropriate dosing, significantly reducing cardiovascular mortality and hospitalization [19]. However, some patients experience side effects such as cough or angioedema, which may limit the tolerability and efficacy of ACEi drugs, even when appropriately dosed. Not all patients are suitable candidates for ACEis due to certain comorbidities (commonly, aortic valve stenosis) or contraindications. For instance, there is a risk of angioedema in patients receiving ACEi, and ARNIs increase the risk of developing angioedema [31].

On the contrary, β-blockers are commonly used in the management of heart failure, but their effects can vary depending on whether they are administered for acute or chronic heart failure, with significant implications for acute decompensated heart failure [70,71]. β-blockers, such as carvedilol, metoprolol, and bisoprolol, significantly improve cardiac function when used chronically as they reduce cardiac workload by slowing the heart rate and reducing contractility [24]. Furthermore, long-term use can help alleviate symptoms such as shortness of breath, fatigue, and fluid retention in patients with heart failure. The use of β-blockers during acute decompensation of heart failure is more complex and requires caution. In cases of acute exacerbation, β-blockers may be temporarily discontinued or adjusted due to exacerbation of symptoms resulting from their negative inotropic effects, which reduce the heart’s ability to pump blood effectively. There is also an additional decompensation risk: the reduction in heart rate and contractility can potentially worsen decompensation during acute episodes, leading to decreased cardiac output and inadequate tissue perfusion [23,70,71]. It is crucial for healthcare providers to carefully assess the patient’s condition when considering the use of β-blockers during acute decompensation. In some cases, temporary discontinuation or dose adjustment may be necessary to ensure hemodynamic stability. Once the acute phase is managed, β-blockers are usually reintroduced and titrated to optimal doses for long-term management [22].

Novelly relied on MRAs, such as spironolactone and eplerenone, which have emerged as valuable additions to HFrEF treatment. They have shown prognostically beneficial effects, reducing mortality risk and hospitalizations in HFrEF patients with symptomatic left ventricular dysfunction. Nonetheless, spironolactone’s non-selectivity as an MRA may lead to side effects such as gynecomastia, impotence, and menstrual disorders. Close monitoring of renal function and electrolytes is necessary due to the risk of hyperkalemia when using MRAs [22-24].

The advent of ARNI drugs, such as sacubitril/valsartan, has offered a pioneering therapeutic option for HFrEF patients. ARNIs combine RAAS inhibition with the prevention of natriuretic peptide degradation, providing a dual mechanism for improved cardiac function. Studies have demonstrated a significant reduction in cardiovascular mortality, overall mortality, and heart failure-related hospitalizations with sacubitril/valsartan therapy compared to ACEi treatment [19,31]. However, being a newer class of medication, long-term safety, and tolerability data may still be evolving. Cost and accessibility could be potential barriers for some patients.

A holistic, novel approach to treating heart failure has investigated the effects of exercise training on patients with permanent atrial fibrillation with reduced ejection fraction. Results showed that exercise-trained groups significantly improved exercise capacity, quality of life, and cardiac function. Moreover, peak oxygen consumption, ventilation per minute/carbon dioxide production, and left ventricular ejection fraction all improved unexpectedly. Additionally, their resting and recovery heart rates decreased, and left atrial dimension, left ventricular end-systolic volume, and left ventricular end-diastolic volume all decreased. Conversely, no significant changes were observed in the no-training group [56,57,59].

It is surprising that MRA and ARNI efficacy competes with that of standardized ACEi dosing, which reduces blood pressure, preload, and intravascular pressure to reduce the workload on the heart [23,28]. This suggests the need for further studies examining the long-term effects of MRA and ARNI on patients, particularly with renal comorbidities, as this lack of long-term potentiation acts as a limitation to the standardization of MRA and ARNI therapies alongside holistic measures such as exercise therapy.

This literature review was aimed at evaluating the existing treatment landscape for HFrEF. The management of HFrEF encompasses a range of therapeutic options, each with its pros and cons. ACEis, ꞵ-blockers, MRAs, and ARNIs have significantly improved prognosis and reduced mortality and hospitalizations. However, carefully considering individual patient characteristics, comorbidities, and preferences is vital in choosing the most appropriate treatment approach. Combining multiple therapies alongside holistic advances such as exercise therapy may provide synergistic benefits, ultimately leading to improved outcomes for patients with HFrEF.

## Conclusions

Heart failure is a prevalent clinical syndrome most commonly caused by ischemic heart disease. HFrEF is diagnosed in 50% of heart failure patients in the United States. It is known to have a mortality rate of 42% in the last five years. The first-line therapy for HFrEF aims at targeting the neurohumoral and sympatho-adrenergic aspects of pathophysiology. Novel pharmacologic treatments including ARNIs, SGLT2 inhibitors, and sGC stimulators, and holistic measures such as exercise-based therapy, have been shown to improve prognosis. Despite this, the existing literature suggests a need for further studies evaluating the long-term effects of MRAs and ARNIs.
